# A possible role for selenoprotein glutathione peroxidase (GPx1) and thioredoxin reductases (TrxR1) in thyroid cancer: our experience in thyroid surgery

**DOI:** 10.1186/s12935-018-0504-4

**Published:** 2018-01-15

**Authors:** Alessio Metere, Francesca Frezzotti, Claire Elizabeth Graves, Massimo Vergine, Alessandro De Luca, Donatella Pietraforte, Laura Giacomelli

**Affiliations:** 1 Department of Surgical Sciences, Umberto I Hospital, “Sapienza” University of Rome, viale Regina Elena 324, 00161 Rome, Italy; 2 Department of Surgery, Columbia University Medical Center, New York, USA; 3 Department of Cell Biology and Neurosciences, Istituto Superiore di Sanità, Rome, Italy

**Keywords:** Oxidative stress, Thyroid cancer, Glutathione peroxidase (GPx1), Thioredoxin reductases (TrxR1), Selenium enzymes

## Abstract

**Background:**

Oxidative stress is responsible for some alterations in the chemical structure and, consequently, in the function of proteins, lipids, and DNA. Recent studies have linked oxidative stress to cancers, particularly thyroid cancer, but the mechanisms remain unclear. Here, we further characterize the role of oxidative stress in thyroid cancer by analyzing the expression of two selenium antioxidant molecules, glutathione peroxidase (GPx1) and thioredoxin reductase (TrxR1) in thyroid cancer cells.

**Methods:**

Samples of both healthy thyroid tissue and thyroid tumor were taken for analysis after total thyroidectomy. The expression of GPx1 and TrxR1 was revealed by Western blot analysis and quantified by densitometric analyses, while the evaluation of free radicals was performed by Electron Paramagnetic Resonance (EPR)-spin trapping technique.

**Results:**

Our results show a decrease in the expression of GPx1 and TrxR1 (− 45.7 and − 43.2% respectively, p < 0.01) in the thyroid cancer cells compared to the healthy cells. In addition, the EPR technique shows an increase of free radicals in tumor tissue, significantly higher than that found in healthy thyroid tissue (+ 116.3%, p < 0.01).

**Conclusions:**

Our findings underscore the relationship between thyroid cancer and oxidative stress, showing the imbalance of the oxidant/antioxidant system in thyroid cancer tissue. These results suggest that either the inability to produce adequate antioxidant defense or an increased consumption of antioxidants, due to the hyper-production of free radicals, may play a crucial role in thyroid cancer.

## Background

The overproduction of free radicals and the consequent increase in oxidative stress lead to irreversible cellular damage, frequently associated with diseases such as atherosclerosis, hypertension, diabetic nephropathy, pulmonary fibrosis, Alzheimer’s disease, and in particular, cancer [1–10]. In thyroid cancer specifically, chemical modifications of DNA structure that are typically induced by oxidative stress, such as the presence of 8-oxo-2′-deoxyguanosine, have been found by microarray and immunohistochemistry analyses [11]. Moreover, high concentrations of glutathione peroxidase (an antioxidant enzyme) and malondialdehyde (a product of lipid peroxidation) were found in the blood of patients affected by thyroid cancer [12]. We recently described an increase of oxidative stress in the blood of patients affected by thyroid cancer, detected by electronic proton resonance measurements, a refined technique to study qualitatively and quantitatively the oxidizing species in human blood. In addition, another interesting finding of our study was the meaningful decrease of oxidative stress observed in all subjects after thyroidectomy [13].

Our previous studies suggest the involvement of oxidative stress in the pathogenesis of thyroid cancer, but the mechanisms remain unclear. Cells regulate the redox state by balancing the generation of free radicals with their elimination by antioxidant systems [14]. A wide array of vitamins, minerals, and other minor compounds are involved these antioxidant systems [15]. In this study, we focused our attention on selenoproteins, a class of proteins containing selenium that seems to play a critical role in the balance of the oxidant/antioxidant systems. In fact, some of them, e.g. glutathione peroxidases (GPxs) [16] and thioredoxin reductases (TrxRs) [17] play a large role in antioxidant defense and in the maintenance of the intercellular reducing conditions. Since increased oxidative stress has been implicated in thyroid cancer, we hypothesized that the impairment of the expression of selenoproteins may be a key factor towards clarifying the relationship between thyroid cancer and oxidative stress. This study elucidates the mechanism involved in the antioxidant/oxidant system in thyroid cancer, focusing the attention on the role played by selenoproteins GPx1 and TrxR1.

## Methods

### Patient recruitment

The study population was recruited in 2014 at the Department of Surgical Sciences of the Umberto I Hospital of Rome. Patients were between 24 and 68 years of age, with thyroid nodules classified as indeterminate (TIR3B), suspicious for malignant (TIR4) or malignant (TIR5) after cytologic examination, according to the “Italian Consensus for the classification and reporting of thyroid cytology, 2014” [18]. The Fine Needle Aspiration (FNA) to obtain samples for the cytological evaluation was performed only on thyroid nodules ≥ 1 cm with suspicious ultrasound (US) features, according to “Sonographic and Clinical Features of Thyroid Nodules and Recommendations for FNA” of the American Thyroid Association (ATA) [19]. Considering the cytological results (Table 1), all patients were candidates for thyroid surgery, according to the ATA 2009, the only one known at the time of the recruitment.

**Table 1:** Classification of the patients, taking into account the preoperative cytological diagnosis (FNA diagnosis), the post-operative diagnosis (histological diagnosis, variant and tumor size), the surgical approach, and the staging (according to the AJCC Cancer Staging Manual 2009, 7th edition)

Case	FNA diagnosis	Histological diagnosis	Variant	Histological size (cm)	Surgical approach	Staging
1	TIR4	Papillary	Classical	0.7	TT	pT1a
2	TIR4	Papillary	Classical	0.6–0.4	TT and level VI	pT1am pN0
3	TIR3B	Papillary	Follicular	1.5	TT	pT1b
4	TIR5	Papillary	Classical	0.8	TT and level VI	pT1a pN0
5	TIR4	Papillary	Classical	1.4	TT and level VI	pT1b pN0
6	TIR3B	Papillary	Follicular	1.2	TT	pT1b
7	TIR3B	Papillary	Follicular	1.5	TT	pT1b
8	TIR5	Papillary	Classical	1.7	TT and level VI	pT1b pN1a
9	TIR4	Papillary	Classical	2.5–1.5	TT and level VI	pT2m pN1a
10	TIR4	Papillary	Classical	1.5–0.9	TT and level VI	pT1bm pN1a
11	TIR4	Papillary	Classical	0.8	TT and level VI	pT1a pN0
12	TIR4	Papillary	Classical	1.8	TT and level VI	pT1b pN1a
13	TIR4	Papillary	Classical	2.8	TT and level VI	pT2 pN1a
14	TIR5	Papillary	Classical	1.4	TT and level VI	pT1b pN1a
15	TIR4	Papillary	Classical	1.6	TT and levels II, III, IV, VI	pT1b pN1a
16	TIR3B	Papillary	Follicular	1.1	TT	pT1b
17	TIR5	Papillary	Classical	2.1	TT and levels II, III, IV, VI	pT2 pN1b
18	TIR5	Papillary	Classical	0.7	TT and level VI	pT1a pN0
19	TIR5	Papillary	Classical	2.8–1.2	TT and levels II, III, IV, VI	pT2m pN1b
20	TIR5	Papillary	Classical	1.3	TT and levels II, III, IV, VI	pT1a pN1b

TT indicates total thyroidectomy, while Roman numerals indicate the location of lymph nodes in the neck

### Surgical treatment

Surgical treatment was planned taking into account the cytological diagnosis, lymph node involvement and the size of the suspicious nodule (both evaluated through US of the neck), age of patients, and a history of prior head and neck irradiation. In cases with a cytologic diagnosis of malignancy (TIR4, TIR5), the patients underwent total thyroidectomy, according to ATA 2009 (recommendation 26). Thyroid lobectomy alone may be sufficient treatment for small (< 1 cm), low-risk, unifocal, intrathyroidal papillary carcinomas in the absence of prior head and neck irradiation or radiologically or clinically involved cervical nodal metastases, as suggested by ATA 2009. However, none of our TIR4/TIR5 patients were candidates for lobectomy. In fact, as previously described, only patients with suspicious nodules ≥ 1 cm underwent FNA and were included in the study. Table 1 reports the histological size of the tumor, measured “ex vivo” after thyroidectomy, which is often different from the pre-operative US measurements. Patients with clinically involved central compartment (level VI) lymph nodes underwent therapeutic central neck dissection along with total thyroidectomy (Table 1, cases 2, 4, 5, 8, 9, 10, 11, 12, 13, 14, 15, 17, 18, 19, 20). Patients with suspicion for lateral lymph node disease (compartments II–IV) on preoperative US or intraoperative exam also underwent lateral neck dissection (Table 1, cases: 15, 17, 19, 20; ATA 2009, recommendation 27). Patients 3, 6, 7 and 16, with indeterminate nodules (TIR3B), preferred to undergo total thyroidectomy, to avoid the possibility of requiring a future surgery on the contralateral lobe as provided by ATA 2009 (recommendation 25b). Informed consent was obtained from all participants. The criteria of exclusion from the study were the presence of debilitating diseases (i.e. advanced stage diabetes, immunological diseases or hematologic disorders), lack of informed consent, and/or lack of authorization form for the processing of personal data. All subjects recruited for the study underwent the following: electrocardiogram, chest X-ray, indirect laryngoscopy, and whole blood sampling. The following blood tests were obtained for each patient: hemochromocytometric analysis, routine chemistry tests, TSH, FT3, FT4, calcitonin, thyroglobulin, anti-thyroperoxidase antibody (TPOAb), and anti-thyroglobulin antibody (TgAb). In order to obtain a comparison between healthy thyroid tissue and cancer tissue in each individual patient, we selected only those patients (candidates for total thyroidectomy) with unilateral suspicious cytological nodules.

### Chemicals and instruments

Antibodies were obtained from the following sources: polyclonal anti-glutathione peroxidase (GPx1) and polyclonal anti-thioredoxin reductase 1 (TrxR1) and RIPA buffer from Sigma–Aldrich (St Louis, MO, USA), monoclonal anti-actin from Santa Cruz Biotechnology (Santa Cruz, CA, USA), peroxidase-conjugated goat anti-mouse and peroxidase-conjugated rabbit anti-goat from Amersham (Arlington Heights, IL, USA). Nitrocellulose paper was obtained from Shleicher & Schuell Bioscience Inc. (Dassel, Germany). Enhanced chemiluminescent kit (ECL kit) and BCA Protein Assay Kit were purchased from Pierce (Rockford, IL, USA). The densitometric analyses of selenium protein were performed using the GS-900 densitometer (Bio-Rad) and the “Image J” Software. Spin trap 1-hydroxy-3-carboxy-pyrrolidine (CPH), 3-carboxy-proxyl radical (CP·) was purchased from ENZO Biochem (Laufelfingen, Switzerland).

### Sampling the thyroid tissue for selenoprotein analysis

A complete histological exam of the thyroid gland was performed after thyroidectomy to characterize the lesions and to obtain the definitive histological classification (Table 1). All patients in which the definitive histological exam revealed, in the uninvolved lobe, an incidental cancer lesion or signs of inflammation or other types of disease were excluded from this study. A sample of tissue containing the thyroid cancer and a sample of the contralateral lobe were taken from the gland at the time of surgery and conserved at − 80 °C for future analysis and measurement of selenoproteins, GPx1 and TrxR.

### Preparation of thyroid gland proteins extract

To obtain a protein lysate on which to detect GPx1 and TrxR1 proteins, each thyroid sample was suspended in 200 µL of phosphate buffer saline and solubilized by incubation for 10 min at 0 °C with an equal volume of 100 mM Tris–HCl (pH 7.5), 0.6 M NaCl, 4% (w/v) Triton X-100, 4% (v/v) sodium deoxycholate, 0.4 mM PMSF, 1 µg/mL leupeptin and 1 µg/mL aprotinin (4× RIPA buffer). The solution was then diluted once with Phosphate Buffer Saline and adjusted to a final volume of 1 mL with 1× RIPA buffer. After centrifugation at 16,000*g* for 30 min at 4 °C, the supernatant, constituted by the thyroid tissue lysate, was collected for the western blot or EPR analyses. The protein content in thyroid tissue lysate was detected using the Pierce BCA Protein Assay Kit, to obtain an equal amount of protein in each sample.

### Western blot analysis

Samples prepared for Western Blot analysis were solubilized in 4× loading buffer, boiled for 5 min, and resolved by Sodium Dodecyl Sulphate–PolyAcrylamide Gel Electrophoresis (SDS–PAGE). Proteins were transferred to nitrocellulose paper at 35 V overnight. Blots were washed with TBS containing 0.05% Tween 20 (TTBS) and blocked with 3% bovine serum albumin in TTBS for 2 h. Washed nitrocellulose filters were incubated overnight at 4 °C with the anti-GPX1 or anti-TrxR1 antibody. After extensive washes in TTBS, the immunoreactive bands were detected by chemiluminescence coupled to peroxidase activity, according to the manufacturer’s specifications (ECL kit; Pierce).

### Densitometric analysis of the selenoproteins GPx1 and TrxR1

In order to measure GPx1 and TrxR1 expression levels, the intensities of specific bands, corresponding to the selenoproteins of interest, were measured. The densitometric analysis of the blots was done using the Image J Software [20]. In brief, the blot images were imported into the software and the contrast was adjusted such that the bands were clearly visible on the blot image. The area around each band was selected and the background intensity was subtracted from the blot image. The intensities of the selected bands were then expressed as a numeric value (arbitrary unit, a.u.) that could be used for carrying out further statistical analyses.

### Electron paramagnetic resonance (EPR)-spin probing analysis of thyroid tissue

Free radicals are highly reactive species with half-lives usually less than 1 ms and are hardly detectable by direct EPR technique alone. This limitation is overcome by combining the EPR technique with spin probing. In brief, an organic compound (in our case 1-hydroxy-3-carboxy-pyrrolidine (CPH), capable of reacting with the radical species present, is added into the study system. The reaction product is a nitroxide radical (CP·), which is much more stable and therefore easily detectable by EPR technique [21]. Spectra were measured on a Bruker ECS 106 spectrometer equipped with a variable-temperature unit (ER4111VT). Samples were drawn up into a gas-permeable Teflon tube with 0.81-mm internal diameter and 0.05-mm wall thickness (Zeuss Industrial Products, Raritan, NJ, USA). The Teflon tube was folded four times, inserted into an open (air) quartz tube, and fixed to the EPR cavity (4108 TMH). The temperature was 37 °C. All ESR spectra were corrected for baseline drift by a linear function and double integrated and simulated using the software supplied by Bruker (ESP 1600 data system). Spectrometer conditions common to all spectra were modulation frequency, 100 kHz; microwave frequency, 9.4 GHz; microwave power, 20 mW.

### Statistical analysis

All data were expressed as the mean (± standard deviation) of at least three measurements and analyzed with the Student’s t test or Two-Way ANOVA with Bonferroni post-test using the statistical software Graph Pad Prism 4.0 (GraphPad Software, Inc., CA, USA).

## Results

### Surgical treatment and outcome

All patients underwent total thyroidectomy. Therapeutic lymph node dissection was performed when cervical lymph node metastases were suspected or shown preoperatively or intraoperatively by the clinical or radiological examination. For all patients, the recovery was uneventful, and discharge was on the third postoperative day. No significant decrease was detected in blood calcium levels after surgery.

### Thyroid histological analysis and staging

Histology of the thyroid tissue demonstrated papillary thyroid cancer in all patients: 16 patients with classical variant (4 with multifocal cancer), and 4 patients with follicular variant. Cervical neck dissection was performed on 15 patients with clinically involved lymph nodes, and lymph node metastases were found in 10 patients. Table 1 summarizes the preoperative cytological diagnosis, the post-operative histological classification, the surgical approach, and the staging.

### Evaluation of GPx1 and TrxR1 expression in thyroid tissue

**Fig. 1:**
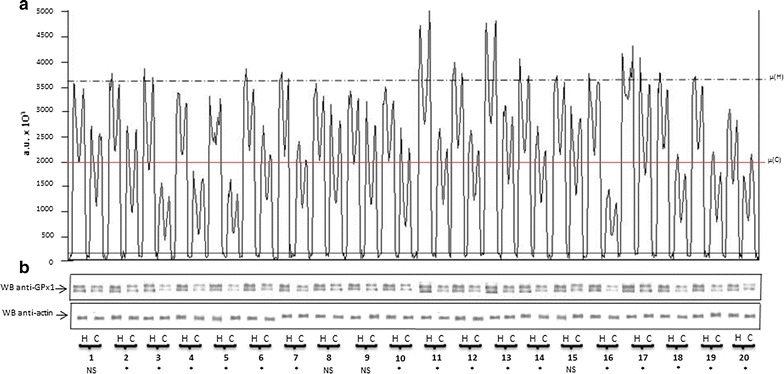
**a** Densitometric analysis of Western blotting reveals a meaningful decrease of the GPx expression (− 44.4 ± 2.8%, p < 0.01) in cancer (red line) with respect to the healthy thyroid tissue (black line). No differences were found between cancer and healthy tissue for the actin expression (data not shown). **b** Western blotting analysis of GPx1 and actin expression in cancer (C) and healthy (H) thyroid tissue. The numbers below identify the patient (see Table 1). *NS* not significant, *p < 0.01)

**Fig. 2:**
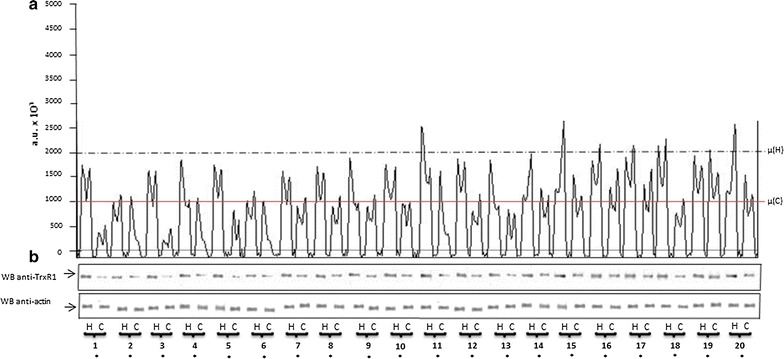
**a** Densitometric analysis of Western blotting shows also in this case a significant decrease of TrxR1 expression (− 49 ± 1.2%, p < 0.01) in cancer (red line) with respect to the healthy thyroid tissue (black line). **b** Western blotting analysis of TrxR1 and actin in cancer (C) and healthy (H) thyroid tissue shows a general reduction of TrxR1 in cancer (C). The numbers below identify the patient (see Table 1). *NS* not significant, *p < 0.01

The overall protein composition of each patient’s thyroid tissue was determined by SDS-PAGE, and the protein content was verified by a specific protein staining (data not shown). The expression of GPx1 and TrxR1 was detected by the appropriate antibodies, able to identify only these two proteins in the thyroid protein lysate. Figures 1 and 2 show the Western blotting results referred to GPx1 and TrxR1, respectively. For each patient, two samples were tested for the presence of GPx1 and TrxR1: one sample from the healthy thyroid tissue and one sample from the cancer tissue. As demonstrated in Fig. 1, the intensity of the band detected by the anti-GPx1 antibody in thyroid cancer tissue was lower for all patients than those detected in healthy tissue. Similar results were obtained using the anti-TrxR1 antibody. In fact, all patients showed a decrease of the TrxR1 expression in cancer tissue compared to the healthy tissue. These findings support the hypothesis that an impairment in the ability of the antioxidant system to counteract oxidative stress may be implicated in thyroid cancer.

### Densitometric analyses of antioxidant enzyme expression

Western blot analyses showed a decrease in GPx1 and TrxR1 expression in thyroid cancer, without a real quantification of the difference. In order to better quantify our results, we performed a densitometric analyses of the bands representing GPx1 and TrxR1 in the western blot. To be sure that these results were not due to a different protein content between the two types of tissue, we decided to normalize our results with respect to actin, a ubiquitous protein present in all eukaryotic cells. Taking all patients together, the densitometric analysis of the GPx1 expression showed a significant lower mean concentration in cancer tissue, compared to healthy tissue. GPx1 expression in cancer tissue (red line, Fig. 1a) was 44.4 ± 2.8% less than the average values detected in healthy tissue (black line, Fig. 1a) (p < 0.001, range + 27 to − 74%). Furthermore, the difference of the GPx1 expression between healthy and cancer tissue, measured for each patient, was statistically significant for 16 of the 20 patients studied (Fig. 1b). The average TrxR1 expression was also decreased in tumor tissue when compared to healthy tissue, a difference of 49 ± 1.2% (p < 0.001, range − 3 to − 63%). Moreover, the mean values of the peaks obtained by the densitometric analysis of TrxR1 expression in thyroid cancer tissue (red line, Fig. 2a) were significantly lower than those measured in healthy tissue (black line, Fig. 2a), 98,965 ± 103,326 vs. 1,963,000 ± 91,242 a.u., p < 0.0001, respectively. Comparing individual patients’ tumor tissue to their own healthy tissue, the difference of TrxR1 expression was statistically significant for everyone (Fig. 2b).

### Free radical production in thyroid cancer detected by EPR-spin probing

**Fig. 3:**
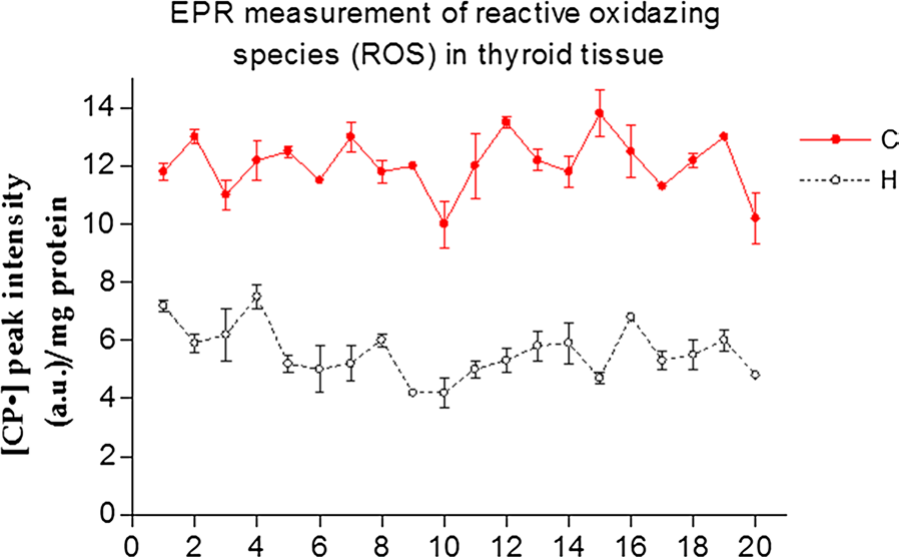
EPR measurement of reactive oxygen species in thyroid tissue. The red line shows the concentration of CP· in cancer tissue (C), while the black line shows the concentration of CP· detected in healthy thyroid tissue (H). The statistical analysis was significant for all patients

As previously described [20] EPR-spin technique has proved very useful in being able to detect free radicals within some human tissues. Using this technique, we detected a significant increase in the production of free radicals in all of the thyroid tumor tissue samples, compared to the healthy tissue of the same patient. In particular, the mean value detected in the cancer tissues was 12.07 ± 0.97 a.u., while that detected in healthy tissues was 5.58 ± 0.89 a.u. p < 0.01). These data indicate, unequivocally, that the process of carcinogenesis is closely related to the physiological mechanisms responsible for the balance between oxidants and antioxidants species (Fig. 3).

## Discussion

It has been suggested that up to 100 selenoproteins may exist in mammalian systems [22], of which up to 30 have been identified by 75Se labeling in vivo [23]. GPx enzymes, known as classical GPx1 (the first selenoprotein identified), gastrointestinal GPx2, plasma GPx3 and phospholipid hydroperoxide GPx4, represent the major classes of functionally important selenoproteins. GPx1 is ubiquitously expressed in many tissues and is present in the cell cytosol and in the mitochondria, where it functions as an antioxidant by directly reducing H_2_O_2_ to water and lipid hydroperoxides to their corresponding alcohol [24]. It may also act as a storage vehicle for selenium, containing 4 selenocysteine residues in a tetrameric structure [25, 26].

Another interesting selenoprotein is TrxR, an enzyme that catalyzes the NADPH-dependent reduction of thioredoxin and therefore plays a regulatory role in its metabolic activity. In fact, thioredoxins are proteins characterized at the level of their amino acid sequence by the presence of two cysteines that act as antioxidants by facilitating the reduction of other oxidized proteins by cysteine thiol-disulfide exchange [27]. TrxR is the only enzyme known to catalyze the reduction of thioredoxin. In brief, the electrons are taken from NADPH via TrxR and transferred to the active site of thioredoxin, which goes on to reduce protein disulfides or other substrates.

Moreover, thioredoxin stimulates proliferation of normal and tumor cells, and is present in high concentrations in tumor cells [28], so an impairment of the TrxR activity may be involved in some forms of cancer. In fact, the expression levels of the cytosolic isoform Trx1 and TrxR1 are linked to tumor aggressiveness, chemo-resistance and to resistance to apoptosis [29–31]. As previously described, numerous types of cancer cells have high levels of free radicals [32–34]. Cancer cells with elevated free radicals production frequently present a compromised antioxidant defense system, which will give rise to an overproduction of free radicals and the consequent increase in oxidative stress, resulting in the impairment of some physiological activities such as cellular metabolism, cellular growth and mitosis [35].

The coupling of the EPR-spin trapping probing technique with CPH can be used to assess the redox pro-oxidant state of cells, both in physiological and pathological conditions. The EPR technique is able to detect the oxidizing species produced and is extremely sensitive to the changes in the redox state [36, 37]. A refined technique like EPR gave us the opportunity to detect the overproduction of free radicals in cancer cell lysate compared to those detected in healthy thyroid tissue.

In this preliminary study, we show that oxidative stress in thyroid cancer is increased compared to that detected in healthy tissues, as suggested by the statistically higher CP· concentration. Moreover, the decrease in antioxidant enzymes GPx1 and TrxR1 in cancer tissue indicates that the antioxidant system in cancer cells is unable to adequately counteract the effects of the free radicals. All patients affected by thyroid cancer showed a reduced expression of these two antioxidants enzymes in tumor tissue, compared to those detected in healthy tissue.

Several different mechanisms are involved in the overproduction of free radicals, resulting in the augmented oxidative stress that afflicts thyroid cancer. As a result, the oxidative stress can only be partially explained by taking into account the defective antioxidant system of seleno-enzymes GPx1 and TrxR1. There are many sophisticated changes in the biology of cancer cells, so to consider only these enzymes as responsible for thyroid cancer is reductive, although our study shows that a contribution of GPxr1 and TrxR1 is likely possible. There are numerous variabilities that we were unable to account for in this small pilot study, such as the size of tumor, lymph node involvement, the multifocality of cancer, or the histotype of cancer. Moreover, our small population of subjects limited our statistical power, though, by comparing healthy vs. tumor samples within the same patient, we attempted to control for many between-patient variables.

## Conclusions

Our findings show, unequivocally, the involvement of oxidative stress in thyroid cancer by demonstrating the imbalance of the oxidant/antioxidant system directly in thyroid cancer tissue. Further studies on the distribution of antioxidant molecules in cancer cells and the characterization of free radical intracellular pathways will be the critical steps in defining the role of the oxidant/antioxidant system in the thyroid cancer. The data obtained in our pilot study push us to further explore the role of seleno-enzymes in cancer. Currently, we are increasing our sample size and performing metabolic studies, with the use of magnetic resonance, to detect changes in metabolites of thyroid cancer cells.

## Abbreviations

GPx1: glutathione peroxidase
TrxR1: thioredoxin reductases
TT: total thyroidectomy
EPR: electron paramagnetic resonance
TPOAb: anti-thyroperoxidase antibodies
TgAb: anti-thyroglobulin antibodies
CPH: 1-hydroxy-3-carboxy-pyrrolidine
CP·: 3-carboxy-proxyl radical
US: ultrasound
